# Ethical Dilemmas and Conventionalism in Healthcare: A Scoping Review

**DOI:** 10.7759/cureus.69693

**Published:** 2024-09-19

**Authors:** Dhruv Ahuja, Puneet Batra, Ojasvi Bhatia, Ashish K Singh

**Affiliations:** 1 Orthodontics and Dentofacial Orthopedics, Manav Rachna Dental College, Manav Rachna International Institute of Research and Studies, Faridabad, IND; 2 Dentistry, Manav Rachna Dental College, Manav Rachna International Institute of Research and Studies, Faridabad, IND

**Keywords:** bioethics, conventionalism, dentistry, ethical dilemmas, healthcare

## Abstract

This review aims to evaluate the current ethical dilemmas faced by healthcare practitioners, especially in dentistry, and analyze how conventionalism with ethical norms influences these challenges. By exploring the balance between evolving healthcare practices and established ethical principles, the review aims to provide insights into the ongoing ethical discussions and dilemmas within the field. A systematic search for relevant articles published between 2000 and July 2024 was conducted across various databases, including Web of Science, PubMed, Scopus, and EBSCO host. Studies that met specified eligibility criteria were included, and data on ethical dilemmas in healthcare and dentistry were assessed. To identify trends and inconsistencies in the existing literature, data extraction and synthesis of the findings were performed. Various ethical conundrums that affect dental healthcare professionals were identified by the literature review. The ethical dilemmas that are revealed through this are substandard dental care, inadequate treatment, inadequate sterilization, identity-related dilemmas, overtreatment, and conflict between truthfulness and beneficence. The review highlights common ethical dilemmas in healthcare, underscoring the need for improved resource management, patient communication, and consistent ethical practices.

## Introduction and background

In the medical profession, although clinical knowledge and technical skills are fundamental, each decision demands a holistic approach. Ethical frameworks, moral considerations, and legal obligations are vital factors that profoundly impact the practice and duties of a healthcare professional [1]. In the modern era, a range of principles has helped shape ethical codes for healthcare professionals. During the early stages of the COVID-19 pandemic, when resources were scarce and knowledge about the virus was limited, healthcare workers were confronted with challenging decisions that often conflicted with their personal ethical and moral values. Such conflicts could lead to negative psychological outcomes, including what is known as moral injury [2]. The primary objectives of ethics are to distinguish between right and wrong and to remain committed to acting on the decision. Healthcare professionals must develop both intellectual and emotional capacities to build ethical readiness, enabling them to make sound decisions during crises such as public health emergencies, natural disasters, or situations involving scarce resources. This preparedness is essential for navigating the complex ethical challenges that arise in such high-pressure environments [3,4].

Dentistry encompasses a broad scientific discipline, integrating preventive, therapeutic, and cosmetic aspects of care. Ethical dilemmas and concerns are inherent to the field, as they frequently present situations with diverse ethical considerations and complexities [5,6]. Dentists face ethical challenges at multiple stages of their practice, starting from the initial oral diagnosis through to the formulation of treatment plans. These concerns arise as they navigate complex decisions about patient care, balancing clinical judgment with ethical considerations at every step [7]. Beyond clinical decisions, dentists also encounter ethical issues in practice management and patient communication. It is essential for dentists to be familiar with fundamental ethical guidelines to handle these situations appropriately and avoid potential pitfalls. Effective communication and a well-defined analytical approach are crucial for evaluating ethical dilemmas. Dentists must be knowledgeable about the four foundational ethical principles: respect for autonomy, beneficence, non-maleficence, and justice. These principles are "prima facie," meaning they are generally obligatory unless they conflict with other moral principles [8].

The evolution of ethical concepts in healthcare, including dentistry, has moved from traditional practices to contemporary standards that emphasize patient autonomy, informed consent, beneficence, non-maleficence, and justice. This shift has introduced various ethical dilemmas, such as maintaining confidentiality, securing informed consent for complex procedures, making end-of-life care decisions, handling treatment refusals, and managing the allocation of limited resources.

Hence, this review aims to evaluate ethical dilemmas in healthcare, especially dentistry, within the context of traditional to modern practices and identify specific areas where conventionalism falls short, contribute to a nuanced understanding of the ethical landscape, and suggest potential solutions or alternative approaches.

## Review

Material and methods

The review accessed authoritative scientific databases, including PubMed Central, Web of Science (WoS), Scopus, and EBSCOhost. Search terms were selected to cover a range of issues related to ethical dilemmas in healthcare, focusing on ethics, ethical dilemmas, healthcare, and dentistry. The review specifically focused on ethical dilemmas and conventionalism in healthcare. Following the Population, Intervention, Comparison, and Outcome (PICO) framework, the review question was specifically designed to investigate the population (healthcare professionals facing ethical dilemmas), intervention (implementation of ethical decision-making frameworks), comparison (conventional approaches to ethics in healthcare), and outcome (effectiveness in resolving ethical issues and improving patient care outcomes). The formulated research question was "Do dental healthcare professionals face ethical dilemmas, and how does the use of structured ethical decision-making frameworks compare to conventional ethics practices in terms of resolving these ethical issues and improving patient care outcomes?"

Search Strategy

**Figure 1:**
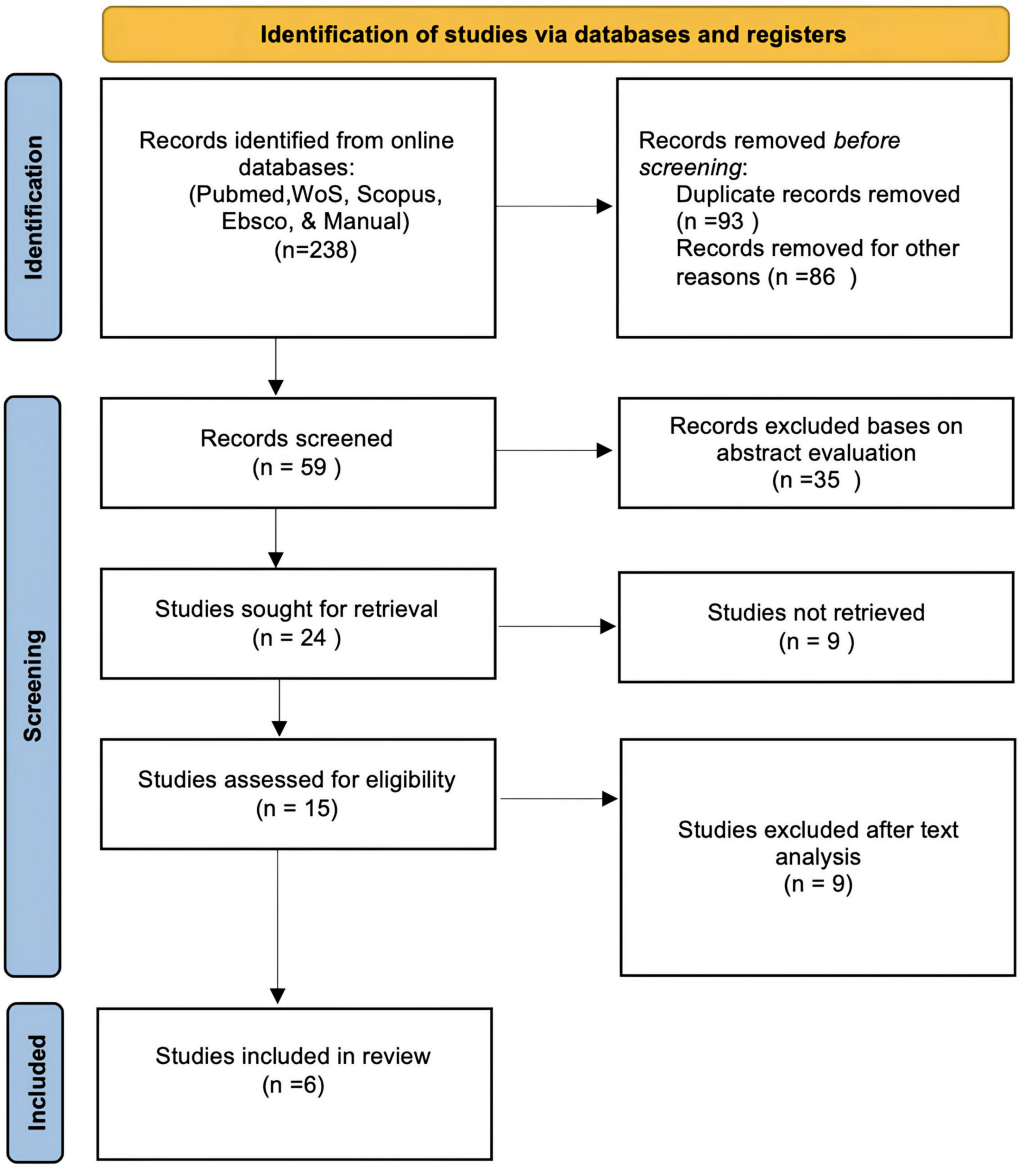
PRISMA flow chart: identification of studies through different databases PRISMA: Preferred Reporting Items for Systematic Reviews and Meta-Analyses

A thorough electronic search was performed across several databases, including WoS, PubMed, Scopus, and EBSCOhost, covering the past 24 years. This search aimed to identify all relevant articles that met the predefined inclusion criteria. Additionally, reference lists from selected articles were manually reviewed to capture any pertinent studies not identified through the electronic search. The search was conducted by two examiners (D. A. and O. B.) in July 2024. Medical Subject Headings (MeSH) terms were used in conjunction with “AND” and “OR” to structure the search strategy as ([ethics]) OR ([ethical dilemmas]) OR ([bioethics] OR [biomedical ethics]) AND ([healthcare]) OR [health]) AND ([dental]) OR ([dentistry]). A two-stage screening process was implemented to identify relevant studies. Initially, titles and abstracts were assessed independently by both researchers (D. A. and O. B.) against the established inclusion and exclusion criteria. Subsequently, full texts of potentially relevant articles were retrieved and evaluated independently by both researchers. Any disagreements were resolved by a third researcher (P. B.) (Figure 1).

Eligibility Criteria

To identify relevant research, this review focused on full-text, English-language studies that address ethical dilemmas in healthcare or dentistry. Included studies explored the intersection of ethical dilemmas with conventional practices within these fields. The eligible studies encompassed empirical research designs, including qualitative, quantitative, and mixed methods approaches. Studies were excluded if they did not clearly address the topic, had methodological flaws, or lacked essential data. Additionally, case reports, letters, and short communications that were irrelevant to the review's goals or potentially influenced by confounding factors were not considered.

Data Extraction and Synthesis

A rigorous approach involving two reviewers was employed to ensure the selection of relevant studies. Abstracts that did not provide adequate information for inclusion were excluded. Additionally, the reference lists of the selected articles were manually reviewed to identify potentially relevant studies that were not captured in the initial database search. After the selection process, data from the included studies were independently extracted by two researchers (D.A. and O.B.) using a standardized format, and the data were compiled into an Excel spreadsheet for subsequent analysis.

Results

The selection process employed a Preferred Reporting Items for Systematic Reviews and Meta-Analyses (PRISMA) flow diagram to depict the study-selection procedure. Following the removal of duplicates and irrelevant abstracts from the databases, 59 studies were subjected to initial screening by two independent reviewers. Any disagreements regarding inclusion were resolved through discussion to achieve consensus. Ultimately, six high-quality scientific studies that aligned with the review's objectives were selected for further analysis (Figure 1).

Characteristics of Included Studies

The electronic search, guided by the PICO framework, identified six relevant studies. This narrative review adopted a rigorous approach, avoiding simplifications in data analysis. We focused on the latest research specifically investigating the ethical dilemmas in health care, focusing on dentistry. Table 1 summarizes the study design/type, related healthcare sector, and ethical dilemmas with key findings from the included studies. It is important to acknowledge that due to the limited data, a formal risk of bias analysis was not performed. The inherent nature of narrative reviews also suggests a potential for bias in the selected studies.

**Table 1:** Literature review on ethical dilemmas in healthcare

S. No.	Author	Study design/sample	Healthcare sector	Ethical dilemma	Inference
1.	Kang et al. (2024) [9]	Narrative inquiry, Sample size: 106	Medical	Identity-related dilemma	The "identity-related dilemma" emerged from the complex role of medical students, who must navigate the expectations and responsibilities associated with being both learners and emerging professionals. This dual identity often creates tension as students balance their educational objectives with the professional standards expected of them.
2.	Antoniadou et al. (2023) [10]	Questionnaire-based survey, Sample size: 133	Dental	Being confronted with inadequate treatment from other colleagues	Dental students frequently face the challenge of having to adjust or reduce their treatment plans due to financial limitations. This constraint often forces them to make difficult decisions about the scope and quality of care they can provide, which may impact patient outcomes and their own educational experience. Balancing cost constraints with the need for comprehensive treatment becomes a significant hurdle in their training.
3.	Holden et al.(2021) [11]	Interview-based qualitative research, Sample size: 20	Dental	Overtreatment	In dentistry, many practitioners encounter the issue of excessive treatments performed by their colleagues. This problem arises when dental procedures are conducted beyond what is necessary or appropriate, often leading to concerns about the ethical implications and potential impacts on patient care and trust.
4.	Kemparaj et al. (2018) [12]	Interview-based qualitative research, Sample size: 25	Dental	Inadequate sterilization and waste management in dental clinics	Dental clinics frequently involve the challenge of ensuring thorough sterilization to maintain patient safety while also managing the environmental impact of waste generated by these processes. Balancing these priorities requires careful consideration of both the health benefits of rigorous infection control and the need for sustainable waste disposal practices.
5.	Priyanka et al. (2016) [13]	Cross-sectional study, Sample size: 135	Dental	Conflict between the principles of truthfulness and beneficence	Ethical dilemmas frequently involve determining which ethical principle should take precedence in a given situation. This decision-making process requires careful evaluation of conflicting values and priorities as professionals weigh the significance of each principle to arrive at the most balanced and justifiable course of action.
6.	Porter et al. (2002) [14]	Questionnaire-based survey, Sample size: 499	Dental	Substandard care by other dentists	Despite the guidance provided by codes of conduct, dentists frequently encounter difficulties in dealing with instances of inadequate treatment conducted by their peers. This challenge often involves navigating the complexities of peer relationships and professional dynamics, even when the standards for practice are clearly outlined.

Synthesis of Result

The studies on ethical dilemmas in healthcare reveal a variety of complex challenges faced by professionals. It highlights major ethical issues, particularly inadequate treatment, which was frequently discussed. The synthesis provides a comprehensive overview of current research and focuses on identifying and addressing these ethical concerns effectively. Inadequate treatment was identified as the most prevalent ethical dilemma across the reviewed studies. This issue involves patients receiving insufficient care due to systemic constraints, resource limitations, or provider shortcomings. Contributing factors include understaffing, lack of access to necessary treatments, and discrepancies between clinical guidelines and individual patient needs [10]. Kemparaj et al. in 2018 showed that inadequate sterilization and improper waste management pose serious ethical issues. Failure to effectively sterilize instruments or manage waste can compromise patient safety and public health [12]. Kang et al. in 2024 revealed identity-related dilemmas involve being negatively impacted by the student-doctor's status. This theme can be divided into two sub-themes: first, "exclusion from learning opportunities," where medical students are overlooked by staff, nurses, and patients due to a lack of recognition of their role. In some cases, identity issues also arise even when participation is permitted, as students may be seen as "pretending to be doctors" to facilitate their practice [9]. Evidence from the literature also showed healthcare professionals often encounter ethical dilemmas when conflicting principles, such as truthfulness versus beneficence, arise. Balancing a patient’s right to be informed about all treatment options with the duty to provide the best care creates moral tension. Resolving these conflicts requires careful consideration and ethical sensitivity to uphold both principles effectively [13]. Studies also depicted healthcare professionals experience ethical sensitivity and moral distress when making complex patient care decisions. Conflicts between clinical guidelines, patient needs, ethical principles, and systemic constraints can cause significant emotional strain. Balancing professional duties with personal values often exacerbates these ethical dilemmas [13,14] and dentists frequently face the ethical issue of overtreatment, where unnecessary procedures may be recommended due to financial incentives, patient demands, or defensive practices. To address this dilemma, dentists must balance comprehensive care with ethical responsibility, prioritizing patient well-being, and avoiding unnecessary interventions [11].

Discussion

Healthcare professionals encounter ethical dilemmas in various settings, such as balancing patient autonomy with medical advice and managing resource allocation between emergency and routine care. These dilemmas, present in hospitals, private practices, and public health clinics, require careful consideration of patient welfare, professional integrity, and systemic constraints. Addressing these challenges demands context-specific strategies, clear ethical guidelines, and continuous reflection.

Ethical dilemmas in healthcare differ widely depending on the setting, each with its own unique challenges. Our review indicates that according to Kemparaj et al. [12], the issue of inadequate sterilization and waste management is notably prevalent. This dilemma, common in both dental clinics and hospitals, underscores the importance of stringent infection control and proper biohazardous waste management to ensure patient safety. Dentists must adhere to rigorous infection control protocols and environmental regulations to safeguard patient well-being and uphold ethical standards in their practice.

The findings from the review also highlight that dentists may encounter dilemmas related to overtreatment, as noted in studies by Porter and Grey [14] and Antoniadou et al. [10]. These issues involve recommending unnecessary procedures and balancing professional advice with patient expectations. Additionally, Noh and Kim [15] describe the ethical dilemma of moral distress, particularly prevalent in long-term care facilities such as nursing homes. This dilemma involves complex issues such as end-of-life care, managing pain, and respecting the wishes of residents with diminished capacities, requiring careful attention to ethical principles and practical considerations. Similarly, emergency settings present ethical challenges related to triage and informed consent under pressure. Healthcare professionals often have to make swift decisions about who receives immediate care based on severity, sometimes without the chance for thorough patient discussions, which raises concerns about fairness and consent.

According to this review, the studies conducted by Holden et al. [11] and Priyanka et al. [13] address critical ethical dilemmas in dental practice. Holden et al. [11] highlight issues related to overtreatment, where dentists may face ethical challenges in recommending unnecessary procedures due to external pressures or misaligned incentives. Similarly, Priyanka et al. [13] explore the ethical tension between professional recommendations and patient expectations, emphasizing the difficulty dentists face in balancing their clinical judgment with the desires of their patients. Both studies underscore the need for dentists to navigate these dilemmas carefully to maintain ethical standards and ensure patient welfare. In primary care, ethical dilemmas often arise when managing chronic diseases and preventive care while respecting patient autonomy. For instance, doctors may encounter challenges when patients refuse vaccinations or preventive treatments, creating a conflict between honoring patient choices and promoting public health benefits. This issue parallels the ethical concerns discussed in the studies by Holden et al. [11] and Priyanka et al. [13], where the balance between professional recommendations and patient expectations is similarly contested. Both situations highlight the ongoing struggle to reconcile individual patient preferences with broader health considerations, underscoring the need for thoughtful ethical decision-making in healthcare.

The evolution of ethical concepts in dentistry reflects a significant shift from traditional to modern standards. Historically, patient autonomy was less emphasized, with dentists making decisions based on their judgment rather than actively involving patients. Modern practices now prioritize patient autonomy through informed consent, ensuring patients have all the necessary information to make well-informed decisions about their treatment. Beneficence and non-maleficence once focused mainly on technical success, are now considered in a broader context. Beneficence involves not only achieving clinical success but also enhancing overall patient well-being and quality of life. Non-maleficence includes understanding the psychological and long-term impacts of treatments. Professional integrity has also evolved from adherence to clinical standards to a greater emphasis on transparency and accountability. Modern standards require dentists to follow strict ethical guidelines, avoid conflicts of interest, and maintain honesty in patient communications and billing practices (Table 2).

**Table 2:** Modern-day ethical controversies and dilemmas in dentistry

Ethical dilemmas in dentistry	
Over-treatment and financial exploitation:	Financial incentives in dentistry can result in over-treatment and unnecessary procedures, raising ethical concerns. This underscores the need for transparency and a focus on patient-centered care.
Informed consent and patient autonomy:	Ensuring informed consent is challenging, especially with complex cases or limited health literacy. Clear communication and avoiding coercion are essential to uphold patient autonomy.
Technology and privacy:	Digital records in dentistry pose risks to patient privacy and data security, including breaches and misuse. Robust security measures are essential to protect patient data.
Cosmetic dentistry and ethical boundaries:	The rise in cosmetic procedures raises ethical concerns about marketing and patient pressure, potentially overshadowing essential treatments. Balancing patient preferences with clinical necessity is crucial.

In summary, traditional dental ethics centered on professional judgment and clinical results, whereas modern standards emphasize a patient-centered approach that addresses issues like informed consent, financial incentives, and data privacy. Current debates highlight the difficulty of applying these evolving principles while balancing patient welfare with technological and financial pressures. To navigate these challenges, dental professionals can rely on ethical decision-making frameworks, consult with peers, pursue ongoing education, demonstrate empathy, and advocate for patient's rights, ensuring the delivery of high-quality, ethical care.

**Figure 2:**
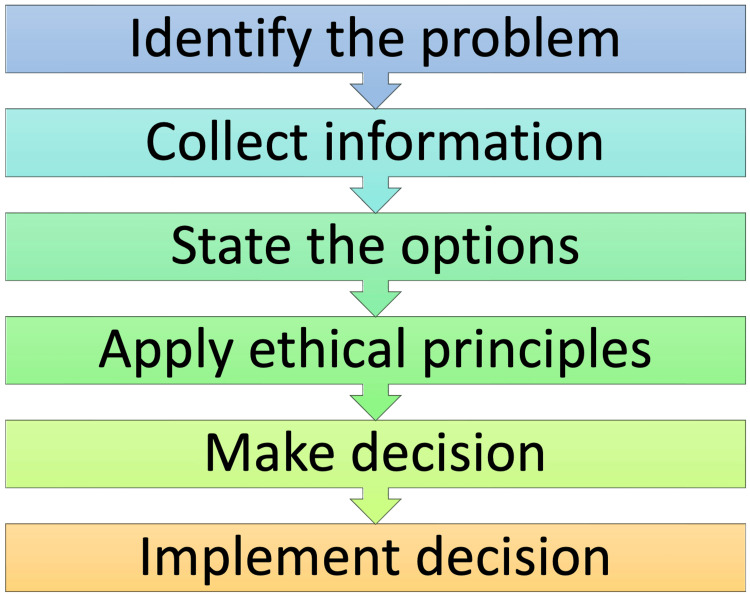
Steps to solve an ethical dilemma Image Credit: Dr. Dhruv Ahuja

Ethical dilemmas emerge when individuals must prioritize one ethical principle over another or when personal values clash with established professional standards. Each healthcare context necessitates a tailored approach to ethical decision-making, considering the specific needs, limitations, and principles relevant to that setting (Figure 2).

Futuristic scope

Future research on ethical dilemmas in healthcare will focus on the complexities introduced by AI, personalized medicine, and data privacy. As technology progresses, new challenges related to consent, equity, and transparency will arise. Addressing these issues will require innovative ethical frameworks and adaptable policies to ensure fair patient benefit. Continued research is essential to tackle these evolving challenges and to ensure that ethical guidelines stay relevant in a rapidly changing healthcare environment [16-18].

Limitations

A significant constraint is the skewness of study results, which can limit their generalizability. Variability in research methodologies and ethical frameworks further complicates drawing consistent conclusions. Conventionalism may lead to relativism, justifying harmful practices accepted within certain cultures and lacking a clear framework for resolving ethical conflicts, especially when cultural norms are disputed or when choices deviate from societal expectations.

## Conclusions

The review of ethical dilemmas in healthcare, especially in dentistry, reflects broader systemic issues that compromise the delivery of optimal care. The significant ethical concerns identified include inadequate treatment, inadequate sterilization and waste management, identity-related dilemmas, conflicts between ethical principles, and over-treatment. To address these issues effectively, ongoing efforts are needed to enhance resource management, improve patient communication, and ensure the consistent application of ethical practices across diverse healthcare settings.
